# *Toxoplasma gondii* and suicidal behaviour: discovery, cross-diagnostic confirmation and pathway exploration

**DOI:** 10.14411/fp.2025.025

**Published:** 2025-09-05

**Authors:** Teodor T. Postolache

**Affiliations:** 1 Mood and Anxiety Program, Division of Biological Psychiatry, Department of Psychiatry, University of Maryland School of Medicine, Baltimore, MD, USA; 2 Mental Illness Research, Education and Clinical Center (MIRECC), Veterans Integrated Service Network (VISN) 5, VA Capitol Health Care Network, Baltimore, MD, USA; 3 Veterans Health Administration, Rocky Mountain Mental Illness Research Education and Clinical Center (MIRECC), Veterans Integrated Service Network (VISN) 19, Aurora, CO, USA

**Keywords:** suicidality, suicide risk factors, depression, impulsivity, aggression, sleep impairment, frailty, suicidal self-directed violence

## Abstract

Our team’s discovery of the link between chronic “latent” infection with *Toxoplasma gondii* (Nicolle et Manceaux, 1908) and suicidal behaviour, and our subsequent cross-diagnostic confirmatory work and mechanistic extensions, evolved from our neuroimmunology studies on affective and behavioural dysregulation exacerbated by allergic sensitisation and allergen exposure. Another root was studying behavioural changes and cytokine gene expression in the brain of rodents sensitised and exposed to aeroallergens. We “piggy-backed” our project funded to study coupling between aeroallergen sensitisation and exposure in patients with recurrent mood disorders, by measuring *Toxoplasma gondii* (*T. gondii*) antibodies in existing samples, and found associations between IgG serointensity and past suicide attempts. Successively, we then reported significant associations between *T. gondii* seropositivity and/or serointensity and suicidal behaviour in patients with schizophrenia in Germany, recent attempters in Sweden, and longitudinally in a cohort of Danish mothers. In the Danish mothers the exposure to *T. gondii* preceded self-directed harm and violent suicide attempts; the association was stronger with higher serointensity strata demonstrating a dose-effect. Furthermore, we identified links between *T. gondii* IgG and suicide endophenotypes aggression and impulsivity in both individuals with no history of mental illness, and in patients with intermittent explosive disorder. We also found associations between *T. gondii* and risk factors of suicidal behaviour such as hopelessness and anhedonia in the Amish, depressive symptoms in pregnant women and women Veterans, frailty in older adults, and cognitive deficits in patients with bipolar disorder. Recently, we reported positive associations between *T. gondii* IgG serointensity with suicidal ideation, impulsivity, depression scores, and daytime dysfunction due to sleep problems in US Veterans who previously attempted suicide. *Toxoplasma gondii* emerged rather unexpectedly and then took over a considerable proportion of our neuroimmune research portfolio. It satisfied both intellectual appetites, and brought celebrations of discovery, with three systematic reviews and meta-analyses published to date, and a substantial majority of primary articles confirming our initial observations. *Toxoplasma gondii* also brought considerable frustrations, such as initial grant application setbacks, inability to completely demonstrate causality and, so far, prophylactic and therapeutic impotence for mental health applications in general. While we do not have, as of today, effective and safe treatments for *chronic* toxoplasmosis with demonstrated mental health benefits in immunocompetent hosts, there are reasons to be optimistic regarding future discoveries. These may include vaccines, novel medications using *in silico* exploration with biological confirmation, trials of reactivation prevention, as well as identification and targeting of mediating mechanisms. Yet the most justified reasons for optimism are the potential to apply machine learning (ML) and artificial intelligence (AI) methodologies to big data with a focus on interaction and causal inference. These novel approaches, utilising ML-weighted models that emulate randomised trials in electronic medical records, have the potential to reveal not only if *T. gondii* elevates risk and to what extent, but also for whom specifically, under which demographic, clinical and physiological circumstances, and what factors, or combinations thereof, might mitigate this risk.

I have been invited to write this autobiographical narrative based on my collaborative work that uncovered and established associations between chronic infection with *Toxoplasma gondii* (Nicolle et Manceaux, 1908) and various aspects of self-directed and interpersonal violence, across diagnostic categories. As a clinical psychiatrist and researcher specialising in mood disorders, anxiety, clinical chronobiology and suicide risk, I have maintained an active patient practice since completing medical school many years ago. As a researcher, I have been investigating how both natural and built environments – physical, chemical, and biological – influence brain structure and function through altering chronobiological, endocrine and immune pathways.

*Toxoplasma gondii* came into my life unexpectedly, “piggybacking” my pre-existing research project in triggers of inflammation leading to the exacerbation of certain risk factors for suicide and suicidal behaviour. In time, it became one of the top interests in my team’s research portfolio. One media interviewer once asked me “Professor Postolache, weren’t you concerned that switching your research towards the idea that a parasite makes some people kill themselves might compromise your scientific career?” I see how this may have been the case for several of my predecessors in behavioural health who were truly pioneers (two of them publish autobiographic articles in the same issue of Folia Parasitologica: Torrey 2024 and Flegr 2025).

I have been aware about the initial reactions of disbelief and even ridicule for their discoveries in professional circles, which was not dissimilar from the “welcome” Louis Pasteur received from the medical establishment. At the time I came to this field there were already pioneering studies that strongly established associations in schizophrenia (summarised in the biographic review by Torrey et al. 2024) and human personality (summarised in the biographic review by Flegr 2025), as well as animal studies demonstrating behavioural aberrations in *T. gondii*-infected rodents (as detailed in the same issue of Folia Parasitologica in the biographic review by Vyas 2024).

Moreover, it is not only important where one lands, but also from where one comes from. It is important to understand that I came to the idea that a neurotropic parasite may cause brain changes that lead to suicidal behaviour from studies on aeroallergens and suicide. In other words, from a field in which we were testing the hypotheses that the romantic life of trees may cause certain individuals (i.e., those sensitised to pollen, and with genetic and developmental risk factors for suicide) to kill themselves. Presented as such, certain people appeared to believe that those hypotheses navigated a course between the ‘far-fetched’and the delusional. Thus, that was of course a much riskier line of research, which, as I was jokingly telling my trainees, could lead not only to professional ridicule, but even an involuntary admission on a locked psychiatric unit.

Luckily, our associations between aeroallergen exposure with affective and behavioural dysregulation were replicated macro-epidemiologically, supported clinically and through our animal and *post-mortem* studies, and thus, made an involuntary admission less likely. In what concerns, *T. gondii* slowly took over the place of aeroallergens in our research. In time, our allergen articles were replicated by several independent studies using similar methods in other settings, and our *T. gondii* suicidal behaviour associations receiving multiple metanalytic confirmations. This contributed to our research on the natural environment and suicide risk to gain wider acceptance.

## Suicidal self-directed violence (SSDV)

Suicidal behaviour, also named suicidal self-directed violence (SSDV), as a nomenclature harmonisation effort by the Centers for Disease Control (CDC), encompasses both fatal suicide and non-fatal suicide attempts. Suicide represents death caused by self-directed injurious behaviour with any intent to die as a result of the behaviour. If the presence of intent is not clear the more encompassing term self-directed violence (SDV) can be used. A positive suicide intent is critical for classifying self-directed violence as being suicidal. Suicide intent could involve any significant possibility of dying as a result of the behaviour – it does not have to be anywhere close to 100%. While suicidality is most often understood as relating to suicide ideation, intent and plan (but not behaviour), some researchers use the term to encompass both ideation and behaviour. For self-harming behaviour with no intent to inflict death, the term nonsuicidal self-directed violence could be used, also called nonsuicidal self-harm.

SSDV is influenced by multiple interacting factors (Turecki and Brent 2016, Lutz et al. 2017). These span biological, psychological, social, economic, and spiritual domains encompassing various vulnerabilities and resiliences, precipitants and deterrents, and both aggravating and protective factors, and availability of means (Joiner and Van Orden 2008). The complexity of these interactions, along with the critical role of means availability, necessitates a comprehensive rather than reductionist approach. In the United States (US), suicide consistently ranks among the leading causes of mortality (Centers for Disease Control and Prevention 2023).

### Toxoplasma gondii

The parasitic infection toxoplasmosis, which stems from the protozoan *T. gondii*, first isolated in a common gundi in Tunis in 1908 (Nicolle 1908), represents a major global health concern. Studies indicate that between 8 to 22 percent of the American population carries the infection (Jones et al. 2001, 2003, 2007, Dubey and Jones 2008) with higher rates in rural and farming communities, such as the Old Order Amish (Markon et al. 2020). Between 25 to 33 percent of the world population is infected (Pappas et al. 2009). It appears that environmental factors play a dominant role as compared to heritability, as my colleagues and I have reported in the Old Order Amish (Duffy et al. 2019). *Toxoplasma gondii* substantially affects mortality, morbidity and quality of life in humans, and also the health of domestic animals, wildlife and ecosystems.

*Toxoplasma gondii* sexually reproduces in the gut of felids, the definitive hosts, which excrete massive numbers of oocysts in their faeces (Frenkel et al. 1975). After sporulation, these oocysts are highly resistant to environmental hardship, thus leading to long-term infestation of the environment. After sporulation, oocysts contain infectious sporozoites that are remarkably potent: a single sporulated oocyst is sufficient to cause infection in intermediate hosts (any warm-blooded animal). Once crossing the intestinal barrier of an intermediate host, *T. gondii* spreads and reproduces asexually in the host’s tissues, slowly, including in the brain and the muscle (Miller et al. 1972, Dubey 2016).

**My scientific trajectory towards *T. gondii*** emerged from my prior engagement in the domain linking inflammation and suicidal behaviour (Erhardt et al. 2013, Brundin et al. 2015, Keaton et al. 2019, Neupane et al. 2023). For example, meta-analyses have confirmed associations between C-Reactive protein (CRP) – an inflammation marker commonly used in clinical practice – and both suicidal ideation and behaviour (Miola et al. 2021). My research group was considered among the pioneers in establishing connections between inflammatory processes, their upstream triggers and perpetuators, and suicidal behaviour. Before we began exploring the *Toxoplasma* field, we have demonstrated that it is actually the specificity of coupling between allergic sensitisation and exposure that leads to depressive symptoms in individuals with bipolar disorder exposed to seasonal peaks of aeroallergens, with adjustment for allergy symptom score (Manalai et al. 2012).

Our team was the first to describe the temporal association of the spring peaks of suicide (Postolache et al. 2010) with peaks of aeroallergens (Postolache et al. 2005, Qin et al. 2013) and had already published the first *post-mortem* study examining cytokine gene expression in the prefrontal cortex of suicide decedents. Notably, we discovered elevated levels of allergy-linked Type 2 cytokines in brain regions previously implicated in SSDV, specifically IL-4 in women and IL-13 in men (Tonelli et al. 2008). This led to our rodent studies that confirmed brain cytokines gene expression and behavioural alteration in our rodent studies with intranasal administration of allergens (Tonelli et al. 2009) in previously sensitised and unsensitised animals.

The type 2 cytokines (previously called anti-inflammatory or Th2 cytokines) initially received less attention in human behavioural dysregulation studies compared to their type 1 counterparts. However, more recent research has revealed their similarly important role in brain structure and function as key regulators of social behaviour, learning, memory (Zipp et al. 2023) and neurodevelopment (Barron et al. 2024). At that time, what grabbed my attention was that they are produced in response to allergens and parasites.

My literature search for parasites that infect the brain and are as widespread as suicidal behaviour brought me to *T. gondii*. The striking early publications on host manipulation by the parasite were already there to fully fascinate me (summarised by Vyas 2024). Then I also discovered the published reports on associations of *T. gondii* and schizophrenia reviewed by Fuller Torrey (Torrey 2024) and personality traits reviewed by Jaroslav Flegr (Flegr 2025) in the same issue of Folia Parasitologica. Soon, my hypothesis of *T. gondii* being associated with suicidal behaviour was formed, and the samples on which I tested the aeroallergen sensitisation and exposure in patients with mood disorders in relationship to depression and history of suicide attempt were available for serological analysis.

This led to the very first report of a link between *T. gondii* (in that first article – IgG serointensity) and suicidal behaviour in individuals with mood disorders (Arling et al. 2009). Prior to that article publication, if one would try to search for *T. gondii* AND (suicide OR suicidal) would get positive hits, but those publications invariably would prove not to be about suicidal behaviour in humans, but about suicidal behaviour in cells – i.e., apoptosis. Incidentally (with potential relevance for understanding identifying and targeting mechanisms), *T. gondii* induces apoptosis in certain cells and modulates it in others (Lüder and Gross 2005, Ahmadpour et al. 2023, Su et al. 2023).

The first author of our initial study, Tim Arling, currently a primary care physician in Washington, D.C., was at that time a medical student on an elective research rotation with my group. It was highly unusual at that time for a medical student to be able to coauthor an article in such a brief time as Tim did, and so much more first-author an article. Still, Tim had an outstanding intelligence, both factual and intuitive, dedication, and was an excellent writer. Moreover, “stars” were aligned favourably – with clinical data already in Excel spreadsheets with sufficient remaining samples. Critical intellectual support was received from Robert Sapolsky from Stanford University, and important logistical help was offered by Bob Yolken, who analysed our initial samples at no cost at the Stanley Division of Developmental Neurovirology at Johns Hopkins.

Our December 2009 publication (Arling et al. 2009) opened a new line of inquiry by establishing the first reported connection between *T. gondii* IgG serointensity and suicidal behaviour. We were really proud to be the very first who found this association. We always used “To our knowledge…we were the first), until at some point we dropped that and went to “We were the first to report…” The significance of this finding was soon underscored by two subsequent studies in 2010: Yagmur et al.’s emergency department study (Yagmur et al. 2010) comparing *T. gondii* IgG seropositivity in suicide attempters versus healthcare workers and visitors (published in June 2010), and Lester’s ecological analysis (Lester 2010) correlating national suicide rates with *T. gondii* seropositivity in pregnant women (published in October 2010). Since then many positive and several negative studies have been published, with four systematic reviews and three meta-analyses confirming a significant positive association between *T. gondii* IgG seropositivity and serointensity with SSDV, with no systematic review or meta-analysis reaching a different conclusion (Sutterland et al. 2019, Amouei et al. 2020, Soleymani et al. 2020, Zerekidze et al. 2024).

Following our initial uncovering of the association in mood disorders (Arling et al. 2009), we pursued a systematic investigation of *T. gondii*’s relationship with suicidal behaviour across diagnostic boundaries. This approach was founded on the understanding that suicide risk factors and mechanisms only partially overlap with the presence or severity of often-implicated mental conditions (Xu et al. 2023). We first conducted a study of suicidal behaviour in 1000 patients with schizophrenia. In younger patients with schizophrenia, we found a significant association between *T. gondii* seropositivity and history of non-lethal suicide attempt (OR 1.59, 95% CI 1.06–2.40, *p* = 0.03). Notably, we found no significant associations between suicidal behaviour and the other chronic pathogens *cytomegalovirus* (CMV) and *herpes simplex virus type 1* (HSV-1), or with certain immune triggers (gliadin), thereby suggesting at that time a potential specific role of chronic infection with *T. gondii* (Okusaga et al. 2011a).

One of the limitations of our initial studies, as in most studies that emerged at that time, was that the attempt preceded the blood collection for the serology of *T. gondii*, and the interval of time between the attempt and *T. gondii* IgG determination was highly variable, and often unknown. To address this issue, I initiated two collaborations in Scandinavian countries, one clinical in Lund, Sweden, and one epidemiological investigation nested in the Danish Registers. The first study was a collaboration with Lena Brundin, currently leading a very active clinical neuroimmunology research program of Grand Rapids, Michigan. Our first collaboration involved linking *T. gondii* serology with history of recent suicide attempt among individuals hospitalised for suicide attempts compared to healthy controls in Sweden, and with scores on a suicide rating scale employed in Sweden estimate risk of suicide in suicide attempters (Niméus et al. 2000).

The results were more robust than in previous studies on retrospective suicide attempts. IgG seropositivity and serointensity were significantly higher in suicide attempters versus healthy controls; seropositivity was strongly associated with the scores on the Suicide Assessment Scale (SUAS), at that time, a promising scale in Sweden believed to be able to predict future suicide attempts (Zhang et al. 2012). The importance of the article consisted of the actual shortening time interval between the blood draw and prior attempt, the attempt’s recency, and clinically estimated risk of suicide leading to a psychiatric inpatient admission in all cases. The limitations were the cross-sectional design and the use of healthy controls, but not including psychiatric controls.

## Our collaborative longitudinal study in Danish mothers with *T. gondii* IgG positivity: temporal precedence and dose-response effect

My largest *T. gondii* study to date was based on the collaborative investigation of a cohort of 45,788 Danish mothers. It offered a prospective approach, with *T. gondii* seropositivity status determined before the SDV/SSDV outcome, although a small number of mothers also had pre-existing attempts (Pedersen et al. 2012). This research was a continuation of projects in Denmark that developed with my National Institutes of Health (NIH) R01 grant supported study on my most “risqué” hypothesis – the predictive associations between spring peaks of suicide (Postolache et al. 2010) and seasonal peaks in airborne allergens (Postolache 2005), accounting for certain other seasonal pro-suicidal factors. We also reported links between allergic rhinitis and asthma with suicide (Qin et al 2011) and airborne pollen with suicide, across seasons (Quin et al 2013).

This work was built upon our other spring peaks in SSDV focused findings– including increases in the incidence in upper respiratory virus infections in spring (Okusaga et al. 2011b). This temporally overlaps with a seasonal nadir in 25-hydroxy vitamin D levels in early spring, and an increased rate of Vitamin D deficiency (Zubizarreta et al. 2022), known to contribute to defective microbial immunity (Postolache et al 2020). In sum, these combined factors could lead to more severe acute infections, exacerbation/reactivation of chronic infections with neurotropic potential, as well as excessive and unremitting inflammation. Additionally, from a socio-economic standpoint, increased social demands for productivity and interpersonal interaction – as well as an increased psychological gradient between lower mood energy and elevated expectations associated with the seasonal rebirth of nature – may contribute to this effect as superbly captured in the opening chorus from Act I from Smetana's The Bartered Bride (Postolache et al. 2010).

My article on seasonality of suicide based on Danish Registers includes a post-abstract quotation from “The Waste Land” by T.S. Eliot (“April is the cruelest month…) subtly illustrating the interaction/interplay between reactivated desires, still dormant capabilities, and natural environmental factors that act in concert to increase risk of SSDV.

The piggy-back *T. gondii* study on SSDV in mothers received essential support from Preben Mortensen, founding director of the Danish National Center for Register-Based Research Program in Aarhus, and from William Eaton, from the Johns Hopkins Bloomberg School of Public Health. Study participants were recruited from a cohort of mothers previously enrolled in a *T. gondii* newborn screening initiative. The screening program was conducted between 1992 and 1995 across five Danish counties, representing approximately one-third of Denmark’s total births during the study period. Neonatal blood samples contain IgG antibodies transferred from mothers through the placental barrier. While *T. gondii* infected newborns do not generate their own IgG until 3 months after birth, maternal IgG is detectable in neonatal circulation at birth. Blood was sampled via heel puncture between postpartum days 5–10 and preserved on filter paper and underwent enzyme immunoassay analysis for *T. gondii* specific IgG. A subset of participants (25%, n = 12,740) had first-trimester maternal serum available and used for validation purposes, showing robust correlation with infant IgG levels (spearman ρ = 0.76, *p* < 0.001).

*Toxoplasma gondii* seropositive mothers showed 53% elevation of subsequent SDV versus seronegative mothers (RR = 1.53, 95% CI 1.27–1.85), with risk correlating with antibody levels. Violent suicide attempts displayed a particularly elevated (81%) risk (RR = 1.81, 95% CI 1.13–2.84) (Pedersen et al. 2012). While the risk of death by suicide manifested a substantial elevation of 105% (RR = 2.05, 95% CI 0.78–5.20), the predictive association was not significant, likely due to limited cases of death by suicide among *T. gondii*-positive women (n = 8). The relationship between *T. gondii* infection and SDV appeared stronger in women without documented psychiatric treatment, though this difference lacked statistical significance. This might indicate either undiagnosed psychiatric conditions in women without documented psychiatric treatment, or potential protective effects of psychiatric medications with anti-*T. gondii* properties, such as valproate (Jones-Brando et al. 2003, Fond et al. 2014, Enshaeieh et al. 2021).

Study strengths include its large population-based cohort with 14-year follow-up, socioeconomically diverse sample, and prospectively collected *T. gondii* antibody measurements independent of research aims. This study advanced two key causality criteria summarised in the Flegr (2025) article in the same issue of the Folia Parasitologica (Flegr 2025). First, it established temporal precedence of putative cause (*T. gondii* seroprevalence) before the putative effects (SDV and SSDV). Second, it demonstrated a biological gradient (dose-response relationship), with *T. gondii* IgG levels categorised as the 25th, 50th, 75th and 90th percentiles showing a significant ordinal relationship to self-directed violence risk, increasing from 1 (reference) to 1.91 (95% CI 1.25–2.79).

*Toxoplasma gondii* infection occurs non-randomly, raising the possibility that individuals with underlying mental health issues might face higher infection risks before entering the healthcare system. Research from Norway found that pregnant women had increased infection risk from several behaviours (Kapperud et al. 1996): eating undercooked meat, inadequately washing produce, changing cat litter, and insufficient cleaning of kitchen tools between handling raw meat and other foods. While we were not able to adjust for these specific risk factors, we did *post hoc* address psychiatric history, which may be linked to a degree with some of these behaviours.

One requirement for causality demonstration is that reverse causality possibility must be taken into consideration. Indeed, there is a theoretical possibility that risk factors that representing potential endophenotypes of suicidal behaviour (Gould et al. 2017), with impulsivity and altered decision-making in particular, are contributing behaviours that put people at risk for infection (Markon et al. 2020). This could lead to a possible spurious appearance of a direct causal effect between *T. gondii* and suicidal behaviour. This is especially damning given that while certain human endophenotypes of suicide behaviour could be modeled in animals, there is no animal model for suicidal behaviour *per se* (Gould et al. 2017).

## Elevated *T. gondii* IgG antibodies in relationship to affective, cognitive and behavioural dysregulation

The higher antibody titre categories could be an effect of the virulence and spread of infection, or the tendency to reactivate more often. This may also occur with a more recent infection, or a reinfection with a different strain (serial measurements of IgG and IgM antibodies would be important for this purpose). Certain phenotypes, such as changes in novelty thinking and intelligence, are stronger related to more recent infections (Flegr et al. 2003), suggesting a transient effect. Other phenotypes, however, are related to the duration of exposure, thus with older infections (Flegr et al. 1996, 2000). As in many individuals’ titres tend to diminish in time, certain effects that need time may be associated with lower titres, and even subthreshold titres. Another potential mechanism involves cross-reactivity with other infections and autoimmune disease with innate and acquired molecular mimicry, where the antibodies themselves might directly affect neural tissue. For a more in-depth discussion of this topic, the reader is directed to the Discussion section our article in the same issue of Folia Parasitologica (Postolache et al. 2025).

## Specificity of the associations with *T. gondii* and suicidal behaviour in comparison with other pathogens

### Cytomegalovirus.

In our own study on 950 patients with schizophrenia there was no association between (CMV) IgG antibodies and suicidal behaviour (Okusaga et al. 2011a). At that time, we were naively thinking that we found “the one” neurotropic pathogen predictively related with SSDV. Yet, more recent data suggest that other infectious agents could be predictively associated with SSDV (Dickerson et al. 2017, 2018, Burgdorf et al. 2019). In contrast to the above findings, the team on a study using the Danish biobank and national registers did not find any association between (CMV) and suicidal behaviour (Nissen et al. 2019), while reporting positive associations with Herpes Simplex 1 infection. A study in Finland identified a negative rather than positive association between (CMV) and suicidal behaviour – with other pathogens including *T. gondii* showing no relationship. We need to point out that even though the study was large, it was relatively underpowered for analysing the relatively infrequent events of SSDV (Lindgren et al. 2020).

### Lyme borreliosis.

Clinical insights suggested possible associations between Lyme disease and suicidal behaviour (Bransfield 2017). More recent analyses using the Danish registers identified positive associations between Lyme borreliosis and suicidal behaviour (Fallon et al. 2021). A recent review documented the presence of suicidal ideation and homicidal ideation among individuals with neuropsychiatric manifestations of Lyme borreliosis, persisting after disease recovery (Brackett et al. 2024).

### Other microbial agents.

We have also identified associations between suicidal behaviour and influenza B but not seasonal coronaviruses (Okusaga et al. 2011b), and contributed to two studies using Danish registers that identified associations between multiple infections and death by suicide (Lund-Sørensen et al. 2016) as well as non-fatal and fatal suicide attempts with multiple infections and antibiotic use, in particular broad spectrum ones (Gjervig Hansen et al. 2019). This may suggest that gut bacterial dysbiosis through increased gut wall permeability may lead to low grade inflammation which could elevate the risk for suicidal behaviour.

## Our contribution to uncovering potential clinical conditions partially mediating associations between chronic toxoplasmosis and suicidal behaviour

We investigated potential links between *T. gondii* and suicide risk factors, endophenotypes, precipitating and risk perpetuating factors. Specifically, we identified significant associations between *T. gondii* IgG and certain suicide risk factors such as anhedonia or hopelessness (Wadhawan et al. 2017), depressed or anxious mood (Groër et al. 2011, Duffy et al. 2015), cognitive deficits (Rensch et al. 2024), or endophenotypes of suicidal behaviour (Mann et al. 2009) such as impulsivity (Cook et al. 2015, Coccaro et al. 2016b, Mathai et al. 2016, Postolache et al. 2025) and aggression (Coccaro et al. 2016b, Mathai et al. 2016).

In contrast, when examining potential links with *T. gondii* infection, we were not initially successful in finding convincing associations with a modifiable risk factor for suicidal behaviour – sleep impairment (Ahmad et al. 2017, Corona et al. 2019). Yet, recently, we reported increased daytime sleepiness attributed to nocturnal sleep impairment in relationship to *T. gondii* IgG serointensity in US Veterans who previously attempted suicide (Postolache et al. 2025).

Another substantial link could be the mediation by medical illness of the association between *T. gondii* and suicide. Medical illness has been shown to be significantly associated with both suicide (Østergaard et al. 2024) and *T. gondii* (Flegr et al. 2014). An important, more precise mediators are indicators of disease burden due to medical illness, which manifest strong positive links with suicide (Østergaard et al. 2024). Of relevance, an ecological analysis suggests that *T. gondii* seropositivity accounts for 23% of variability of disease burden in Europe (Flegr et al. 2014).

### Repeat attempts.

The paradox of suicide risk assessment lies in one of its most consistent predictors: while previous repetition of deliberate self-harm (Zahl and Hawton 2004) and suicide attempts (Hawton 1987, Bjarnason and Thorlindsson 1994) indicates increased future risk of suicide, a considerable number of deaths by suicide occur in a sizable proportion of individuals without history of attempts, complicating prevention strategies. However, since our first study (Arling et al. 2009) to the most recent one (Postolache et al. 2025) we were unable to identify a significant association of *T. gondii* IgG with repeat suicide attempt.

In the largest study I participated in, the longitudinal cohort study in mothers in Denmark, for women with a pre-existing history of self-directed violence before giving birth, those who tested positive for *T. gondii* antibodies were 1.54 times more likely to engage in self-directed violence after delivery compared to women who tested negative. However, this finding was statistically “trending” but not statistically significant (*p* = 0.06), likely due to a much smaller sample size relative to women without prior history of self-directed violence (Pedersen et al. 2012).

Yet other investigators were luckier. For instance, the group of Alvarado-Esquivel reported this association twice: one time in a case-control study in psychiatric outpatients (Alvarado-Esquivel et al. 2013) and once in medical/primary care outpatients (Alvarado-Esquivel et al. 2021b). In the later study Alvarado-Esquivel et al. (2021b) reported that the number of prior attempts should exceed three to detect a significant association between repeated attempts and *T. gondii*. An analysis with actual quantification of the number of attempts with a threshold effect was, to my knowledge, not previously undertaken. It might be a low hanging fruit for a colleague to investigate this question in existing data that provide numbers of prior suicide attempts and have already seropositivity and serointensity performed on the sample, or availability of frozen plasma or serum from previous studies not focused on *T. gondii*.

## Alcohol, *T. gondii* and the natural antibiotic peptide cathelicidin

Alcohol use disorder is both a predisposing risk factor and a trigger for SSDV (Pompili et al. 2010, Kittel et al. 2019, Edwards et al. 2020, Holma et al. 2020). *Post-mortem*, *T. gondii* seropositivity was associated with the presence of alcohol in the blood (Samojłowicz et al. 2013, 2017), although no associations were found between *T. gondii* DNA in the brain and excessive alcohol use (Suvisaari et al. 2017). Yet others’ efforts have been successful obtaining positive results, in particular the group of Alvarado-Esquivel in Mexico. Recently, they reported that in individuals with “alcohol consumption” (at least one alcoholic drink every month) *T. gondii* IgG (but not IgM) seropositivity was associated with suicidal ideation, but only in women with history of suicide attempts (Alvarado-Esquivel et al. 2021a). Yet a direct comparison of individuals who consume alcohol with those who do not led to unexpected results, with lower consumption of alcohol in *T. gondii* seropositives (Alvarado-Esquivel et al. 2023). This could be due to pharmacokinetic or pharmacodynamic mechanisms with dopamine production by the parasite in the brain potentially increasing the dopaminergic tone and decreasing the need to rely on alcohol to attain a sufficient hedonic tone in the brain.

A potential “double-hit” hypothesis of both factors interacting as well as increase alcohol or substance use disorder by chronic infection with *T. gondii* have been considered. The dopaminergic production capability of *T. gondii* has been thought through as a potential factor leading to alcohol and substance abuse. However, more recently, an increasing number of studies has pointed out the low-grade chronic inflammation as a consequence of drinking (Adams et al. 2020). Additionally, the perturbation of inflammatory homeostasis secondary to alcohol consumption as both an acute and chronic alcohol use potentially results in a diminished efficacy of antimicrobial defense mechanisms (Chan and Levitsky 2016).

Based on an ongoing project we have preliminarily reported that the antimicrobial peptide cathelicidin (active form LL37) level in the blood is decreased in individuals with alcohol use disorder (Postolache et al. 2024). We had previously identified for the first time, to our knowledge, the first association of a lower expression of cathelicidin in the dorsolateral prefrontal cortex and anterior cingulate cortex in the brain of individuals who died by suicide relative to psychiatric controls who died from non-suicide related causes (Postolache et al. 2020). Thus, it is theoretically possible that a decrease in antimicrobial peptides’ activity, and in particular decreased LL37, may mediate associations between alcohol use and severity of *T. gondii* infection and, in particular, its reactivation, potentially leading to increased SSDV risk. This is one of the hypotheses I would like to be able to test in the upcoming years.

## Biological mediation

Our interpretation of biological, including histopathological, cellular and molecular links, extends beyond the scope of this short biographic review. The immune, hormonal, monoamine, predictive connections between *T. gondii* and suicidal behaviour have been discussed in more depth in our prior publications (Postolache et al. 2021). I will develop, however, the links between *T. gondii* and kynurenine (KYN) pathway, a domain where my collaborators and I have contributed primary data representing a platform for future developments.

## *Toxoplasma*, suicidal behaviour and the kynurenine pathway

Tryptophan, an essential amino acid and its degradation pathway enzymes and metabolites is central to interactions between the microorganism and the host, in particular its immune cells involved in antimicrobial resistance. Certain commensal organisms capable of synthesising tryptophan (TRP) (autotrophs) provide supplementary TRP to complement dietary sources for their host organism. In contrast, many pathogenic microorganisms trigger the host’s indoleamine 2,3-dioxygenase enzymes (IDO) and subsequent catabolic pathway enzymes, resulting in TRP depletion and the production of active metabolites that collectively downgrade the immune system’s antimicrobial functions (Zhang and Rubin 2013). However, numerous intracellular pathogens, being TRP auxotrophs, depend completely on their host organisms for TRP acquisition.

This metabolic dependency makes these pathogens susceptible to the host’s defensive mechanism of depleting local TRP concentrations, thereby restricting pathogen proliferation and virulence (Ren et al. 2018, Costantini et al. 2020). Among mammalian parasites, several species exhibit TRP auxotrophy, with *T. gondii* being one of the most notable examples. *Toxoplasma gondii* triggers IDO-1 activation through pro-inflammatory cytokines, leading to TRP catabolism into KYN. While this serves to deprive *T. gondii* of essential TRP (Pfefferkorn 1984), it also increases KYN metabolites that cross the blood-brain barrier and modulate NMDA receptor function through compounds like quinolinic acid (QUIN) which is excitotoxic, and kynurenic acid (KYNA) which is neuroprotective.

Approximately fifteen years ago I initiated a collaboration with the suicidologist John Mann and his group at Columbia University. Our collaboration identified for the first time a link between suicidal behaviour in individuals with major depression and elevated blood level of KYN (Sublette et al. 2011). KYN is the immediate metabolite of TRP via the IDO-1 pathway, molecule with production upregulated by proinflammatory cytokines and stress (Sublette and Postolache 2012). I remember at that time the conversations with my Columbia collaborators who were focused on serotonin and suicide, and how TRP depletion would make sense. Yet, implicating KYN production beyond TRP depletion, with NMDA agonism by QUIN and NMDA antagonism by KYNA simply appeared to them implausible. I remember the joy of seeing the first email of the Columbia statistician with the results showing that it was indeed the high KYN, and not low TRP, that was significantly associated with suicidal behaviour.

Following up on our first report on the positive association between plasma KYN and suicidal behaviour in individuals with major depression (Sublette et al. 2011) collaborative work with Lena Brundin’s group has focused on neuroactive kynurenine metabolites in the cerebrospinal fluid (CSF) and later, both CSF and blood. The sampling occurred in hospitalised patients recently admitted for suicide attempt, with longitudinal follow up. It identified state-dependent associations between recent suicidal behaviour and high excitotoxic QUIN levels (Erhardt et al. 2013) and low neuroprotective astrocyte produced PIC or KYNA levels (Brundin et al. 2015).

These results led us to evaluate associations with suicidal behaviour of interactions between *T. gondii* and plasma KYN in individuals with schizophrenia (Okusaga et al. 2016). We found that elevated plasma KYN was associated in a nonlinear fashion with history of suicidal behaviour only in seropositive individuals but not those seronegative for *T. gondii*. This cumulative nonlinear effect suggests several potential intertwined biological processes. Considering the immunosuppressive effect of certain kynurenines, it is possible that high KYN will result in a lower immune pressure on the bradyzoites which will reactivate more often and more intently, transforming into tachyzoites and invading other sites, organs and tissues. For this effect of the potential immunosuppressive effect of KYN to become manifest it is important to have a chronic latent infection with potential for reactivation, otherwise the KYN elevation is unrealised.

Additionally, the results of this paper also suggested that inflammation (which activates IDO to shunt serotonin towards the KYN pathway) or KYN levels are not sufficient to be associated with suicidal behaviour in the entire sample of schizophrenia patients. Similarly, in the entire sample *T. gondii* seropositivity was not associated with suicidal behaviour, consistent with negative results of meta-analyses when selecting solely patients with schizophrenia (Sutterland et al. 2019). Our results suggest that despite schizophrenia, including its new onset, being a condition strongly associated with *T. gondii* (Mortensen et al. 2007, Torrey et al. 2007, 2012), suicidal behaviour in patients with schizophrenia is associated with *T. gondii* only in younger patients (Okusaga et al. 2011a), or in those high KYN (Okusaga et al. 2016).

It may be possible that because of the consistent associations of schizophrenia and both *T. gondii* (Mortensen et al. 2007, Torrey et al. 2007, 2012) and alterations of kynurenine pathways (Wonodi and Schwarcz 2010, Plitman et al. 2017) – the individuals with schizophrenia who did not attempt suicide manifested elevations in serointensity and kynurenines sufficiently to blunt the links of *T. gondii* and kynurenine levels analysed individually.

Fifteen years ago, my hopes were high that we would be able to be effective by addressing *T. gondii* etiologically and engaging physiologically the KYN pathway to (fundamentally) prove causality and (clinically) reduce the risk for SSDV. But, alas! Here we are 15 years later, with not much progress, at least for now. In general, we have no effective intervention for chronic toxoplasmosis in immunocompetent hosts, more specifically for helping patients with schizophrenia and other mental illnesses who are positive for *T. gondii* with incomplete response to psychotropics, and we have no currently clinically immediately available pharmacological agents in use to directly target specifically the KYN pathway and chronic toxoplasmosis.

In my clinician scientist experience this is a relative disappointment by comparison to other environmental triggers, perpetuators and exacerbators. For other sources of chronic or reactivating inflammation we have proven interventions. For instance we have dental root scaling and planning, and antibiotics targeting anaerobes, for periodontal disease, intranasal corticosteroids for allergic and infectious acute or chronic rhinosinusitis, pre- and probiotic agents as well as butyrate interventions to reduce intestinal permeability for gastrointestinal pathogens. For herpes 1 and 2 and (CMV) and HIV we have antivirals to prevent and reduce reactivation and for Lyme disease specific antimicrobials and anti-inflammatories.

## *Toxoplasma gondii*, frailty and suicidal behaviour

Another area we have contributed recently to *Toxoplasma* research is frailty in the elderly, recently linked to suicidal behaviour (Kuffel et al. 2023). We have recently reported the first association between *T. gondii* serointensity in seropositives and geriatric frailty (Mohyuddin et al. 2024), resisting adjustment for depression and cognitive deficits. This is particularly relevant because sarcopenia is often featured in frailty, yet the negative effects of the muscular invasion by *T. gondii* have received so far, only cursory interest (Jin et al. 2017). There were no prior articles on *T. gondii* and frailty, although there were several on chronic neurotropic viruses. There is a price to pay for every “first of its kind” excitement. It is much harder to publish. It took us 11 submissions of this frailty article to be able to pass the editorial review. The article ended up being published in a Q1 journal, and complemented existing studies on *T. gondii* and dementia and cognitive deficits as well. It also points towards the muscular component of chronic localisation of bradyzoites, possibly opening both novel pathophysiology and methodology for *T. gondii* research.

## Interpersonal aggression, aggressive personality traits and *T. gondii*

Interpersonal violence and interpersonal aggression are related but distinct concepts. Interpersonal violence refers to the actual use of physical force intended to harm another person or persons. It involves direct physical actions that cause or have a high likelihood of causing injury, harm, disability or death. Examples include hitting, kicking, stabbing or shooting. Interpersonal aggression is a broader term that encompasses both physical and non-physical behaviours intended to harm others, while targets of aggression are motivated to avoid it (Allen and Anderson 2017). It includes verbal aggression (threats, insults, yelling), psychological/emotional aggression (intimidation, humiliation), social aggression (exclusion, rumor-spreading), and physical aggression (which can escalate to violence).

So key differences are: a) severity: violence represents the more severe end of aggressive behaviour; b) physicality: violence is always physical, while aggression can be non-physical; c) scope: aggression is the broader category that includes violence as one form; d) intent and outcome: violence always involves intent to cause physical harm and typically results in injury, while aggression may have different intentions and outcomes. For an overview of the phenomenology and psychobiology of aggression and intermittent explosive disorder the reader is directed to a specialised book chapter (Coccaro et al. 2019).

Aggressive behaviour predicts increased risk of suicide attempts in military service members (Schafer et al. 2022) and civilians (Franklin et al. 2017). The prevalence of aggression, in particular in military populations is concerning, in studies shows that about one-third of veterans exhibit physical aggressive behaviours (such as actions meant to physically harm others) in a given month (Macmanus et al. 2015). Moreover, in any given year, one in ten veterans report incidents of severe violent behaviour, including using weapons against others (Macmanus et al. 2015). Cross-sectional observational studies have demonstrated a significant association between aggressive behaviour and suicidal ideation (Mcglade et al. 2021) as well as between aggression and suicide attempts (Elbogen et al. 2018).

Studies that track individuals over time have found that aggressive behaviour often is manifested prior to suicide attempts, and people who display aggression tend to make suicide attempts with higher lethality (Oquendo et al. 2021). Among active-duty military personnel, individuals who reported aggressive behaviours demonstrated higher rates of suicidal ideation compared to their non-aggressive counterparts (Start et al. 2019). Within a cohort of veterans participating in a PTSD treatment program, the co-occurrence of aggressive behaviour and historical suicide attempts was more commonly reported than the presence of suicide attempts alone (Watkins et al. 2017).

What are the mechanisms by which aggression is related to suicidal ideation and behaviour? One psychosocial mechanism is isolation: aggression leads to interpersonal conflict and social isolation (Rynar and Coccaro 2018, Hammett et al. 2021), and in turn social isolation worsens the risk for suicidal behaviour (Van Orden et al. 2010, Hartley et al. 2018, Calati et al. 2019). Other mechanisms include the history of aggression leading to increased capability for suicide behaviour (Van Orden et al. 2010, Klonsky and May 2015), i.e., blunting of the natural fear of death and dying, and with availability of lethal means, specifically firearms.

Aggression is linked with the firearm ownership, a major suicide risk factor, in both civilian (Sanchez et al. 2020, Clare et al. 2021) and military populations (Heinz et al. 2016). Aggression also leads to increased probability for injury, including traumatic brain injury (TBI), which in turn is associated with increased suicide risk via decreased belongingness, increased burdensomeness and capability to engage in suicidal behaviour (Wadhawan et al. 2019). Finally, alcohol use is positively associated with both aggression (Coccaro et al. 2016a) and suicide attempts (Poorolajal et al. 2016, Bohnert et al. 2017). Although it is likely that there is a component of bidirectionality, the prevailing model is that aggression leads to increased alcohol use which, in turn, increases the risk for attempting suicide (Conner et al. 2003, Conner and Ilgen 2016).

A major issue of previous studies is that they have not controlled prior suicide attempts, which could be confounding or moderating the interaction between all risk factors and aggression and have seldom used mediation analyses. A recent study in active-duty military personnel analysed the putative mediators of alcohol use, social isolation and access to firearms. It found that predeployment aggression predicted suicide attempts at one year postdeployment after controlling for past suicide attempts, with alcohol use (positively) and social network size (negatively) being significant mediators, but not firearm access (Krauss et al. 2024).

## Our studies on *T. gondii* and interpersonal aggression/aggressive personality traits

We evaluated aggression in relationship to *T. gondii* in two extreme samples – the first one was in psychiatrically (perfectly) healthy adults, and the other one in highly aggressive individuals diagnosed with Intermittent Explosive Disorder.

## Our study in *T. gondii* IgG associations with Intermittent Explosive Disorder (IED), and traits of impulsivity and aggression (Coccaro et al. 2013)

The research aimed to investigate the potential relationship between latent *T. gondii* infection and aggressive behaviour, using both categorical classifications and continuous measurements of aggression. We analysed *T. gondii* antibody levels in a study population of 358 adults, comprising three groups: individuals diagnosed with Intermittent Explosive Disorder (IED), psychiatric control subjects with other mental health conditions, and healthy control participants. We enrolled only medically healthy participants. Our study protocol included measurements of multiple behavioural and psychological variables, including aggressive tendencies, anger expression, impulsive behaviours, and emotional states such as anger, depression, and anxiety, in both acute and persistent forms.

Psychiatric diagnoses, including personality disorder diagnoses were made according to DSM-5 criteria using a Structural Clinical Interview (First et al. 2015). We excluded participants who had current substance use disorders, or lifetime history of bipolar disorder, schizophrenia (or other psychotic disorders), or intellectual disability. We categorised our participants into three groups: 110 subjects showed no evidence of psychiatric conditions (designated as Healthy Controls, HC); 138 participants met diagnostic criteria for either syndromal psychiatric or personality disorders but not for intermittent explosive disorder (designated as Psychiatric Controls, PC); and 110 participants fulfilled the diagnostic criteria for intermittent explosive disorder.

Psychometric Measures of Aggression, Impulsivity, and Related Behaviours included the Life History of Aggression (LHA) assessment (Coccaro et al. 1997), which documented participants’ history of actual aggressive behaviours, and the Buss-Perry Aggression questionnaire (BPA) (Buss and Perry 1992), which evaluated aggressive tendencies as a personality trait. The BPA specifically measured both physical and verbal aggression components. To assess impulsivity, we employed two measures: the Barratt Impulsivity Scale (BIS-11) and the impulsivity scale from the Eysenck Personality Inventory-2 (EPQ-2) (Eysenck and Eysenck 1991). Both instruments evaluate an individual’s dispositional tendency toward impulsive behaviour as a personality trait. An important feature of the study was that patients were unmedicated for at least four weeks prior to study assessments.

Our analysis revealed a positive association between *T. gondii* seropositivity rates and/or presence of IED diagnosis, and continuous composite aggression measurements among participants (*p* < 0.05). This association remained significant even after controlling for comorbid psychiatric or personality disorders and was not explained by participants’ depressive or anxiety-related states or traits. Composite Aggression scores remained elevated in *T. gondii* seropositive participants even after adjusting for Composite Impulsivity scores. However, when we adjusted Composite Impulsivity scores for Composite Aggression scores, we found no significant difference. In our binary logistic regression analysis, adjusting for age, we found that composite aggression showed a significant association with *T. gondii* seropositivity (*p* = 0.029), while Composite Impulsivity did not (*p =* 0.773). When we included composite aggression scores in our model, the previously observed relationship between *T. gondii* seropositivity and IED diagnosis (compared to healthy controls) was no longer significant (*p* = 0.675). This suggests that it is aggression that mediates the association between IED and *T. gondii* seropositivity. Additionally, as a secondary analysis, there were higher state and trait anger scores but not anxiety and depression scores in *T. gondii* seropositives (*p* < 0.05).

Several potential mechanisms may explain the observed relationship between *T. gondii* infection and aggressive behaviour. One hypothesis centres on chronic low-grade neuroinflammation triggered by latent *T. gondii* infection, which could influence neurotransmitter systems implicated in aggression. Additionally, *T. gondii* infection may disrupt cortico-limbic neural circuits that regulate impulsive aggressive behaviours.

Several neurochemical pathways may mediate associations between *T. gondii*’s IgG seropositivity with aggressive behaviour. One mechanism involves inflammatory activation of IDO in the KYN pathway, potentially reducing serotonergic while enhancing glutamatergic neurotransmission (Schwarcz and Pellicciari 2002) – systems implicated in impulsive aggression by human studies (Yanowitch and Coccaro 2011, Coccaro et al. 2013). Another candidate mechanism involves increased dopaminergic signaling, which has been linked to aggressive behaviour (Coccaro and Lee 2010). Research demonstrates increased brain dopamine content in *T. gondii* infected mice (Stibbs 1985), and *T. gondii* infected cells exhibit enhanced stimulation-induced dopamine release (Prandovszky et al. 2011).

There is a preponderance of evidence that testosterone levels contribute to a certain degree to interpersonal aggression (Book et al. 2001, Batrinos 2012). While most studies in humans found that individuals seropositive for *T. gondii* have an increased testosterone level relative to seronegatives, some studies reported a decreased level, and other studies found no significant differences, and other studies indicated sex differences in the direction of the association, with a pooled metanalysis confirming higher levels of testosterone seropositive humans, in both men and women (Abdoli et al. 2024). These pooled results were not matched by the non-human animal studies which appeared to be more heterogenous. (Abdoli et al. 2024).

Several limitations warrant consideration. The cross-sectional design precluded inferences of causality or directionality in the observed relationships. Having the patients unmedicated, although it minimised a confounding element, it further diminished the generalisability of the findings. While the finding was a hypothesis driven – thus making me quite enthusiastic in terms of how aggression might mediate the association between *T. gondii* and suicidal behaviour, I was pretty deflated when I found out, *post hoc,* that suicidal behaviour was not significantly associated with *T. gondii* seropositivity in this highly aggressive sample. Similarly, in the US Veterans we did not find an association between seropositivity and suicidal behaviour, and between measures of aggression and *T. gondii* in any subgroup (Postolache et al. 2025). This suggests that interpersonal aggression and suicide could be unrelated to *T. gondii* seropositivity when the samples include a high proportion individuals scoring high on aggressive traits.

Furthermore, there is a certain degree of heterogeneity involved in the association between aggression and suicide risk. In a very recent study in US Veterans (Saulnier et al. 2025) anger/hostility were actually protective, rather than suicide risk elevating. This divergence also appeared in the discordant European versus non-European findings for the mortality data from self-directed versus interpersonal violence (Flegr et al. 2014). Differences in strains, in the form of transmission (oocyst versus tissue cyst-infected, with data suggesting a more severe clinical course for the oocyst), difference in exposure to a rich microbial environment during a critical time of the childhood, the differences in gun availability, religious deterrents, and the “ecological fallacy” may all contribute to this unexpected direction of association.

We have found a lower cortical thickness by MRI in those with positive oocyst antigen but not in those with overall *T. gondii* seropositivity, suggesting a more virulent course, greater neurotropism and potentially a greater immune activation (Postolache et al., unpublished). Additionally, using seroprevalence data from women of childbearing age and extrapolating to both sexes and older ages (Flegr et al. 2014) could introduce measurable artifacts as a result of heterogeneities between countries in terms of sex differences in suicide rates and in *T. gondii* prevalence and virulence, considering in part the potential for sex-dependent differences in the oocyst versus tissue cyst infection, based on reported differences in specific *T. gondii* infection risk factors in men versus women (Markon et al. 2020). Furthermore, identifying differences in associations between socioeconomic variables and *T. gondii* seroprevalence between European countries and non-European countries may create artifacts with or without socioeconomical adjustment. It appears that associations between elements of the aggression continuum, *T. gondii* IgG and SSDV are context dependent, and require longitudinal studies with mixed effects modeling to be better understood.

## *Toxoplasma gondii*, trait aggression and impulsiveness in healthy adults (Cook et al. 2015)

Impulsivity and aggression have been described as endophenotypes for suicidal behaviour (Mann et al. 2009, Gould et al. 2017). For inclusion in the control group (N = 1000), individuals needed to meet two criteria: they HAD TO have no history of suicide attempts and must show no evidence of DSM-IV Axis I or Axis II disorders when evaluated using the Structured Clinical Interview. Blood samples were drawn for all participants and plasma antibodies against *T. gondii* and two other latent neurotropic pathogens (HSV-1 and CMV) measured with ELISA. Participants were assessed with a German version of the Buss-Durkee Hostility and with the Disinhibition subscale of the Sensation Seeking Scale-V for measuring impulsive sensation-seeking.

Our study revealed a gender-specific relationship between *T. gondii* infection and personality traits of aggression and impulsivity (Cook et al. 2015). Female subjects who tested positive for *T. gondii* IgG antibodies demonstrated significantly elevated trait reactive aggression scores (*p* < 0.01), while male subjects showed no such correlation. Additionally, among males (age 60 or below), *T. gondii* seropositivity correlated with higher impulsive sensation-seeking behaviour. The study also investigated potential associations with (HSV-1) and (CMV), but no statistically significant relationships were observed for either pathogen (Cook et al. 2015).

The gender differences were consistent with the pioneering initial studies of Flegr and his group (described in Flegr 2025 in this issue). However, our study’s unique feature is the exclusion of psychopathology, including of personality disorders, using the Structural Clinical Interview for DSM-IV (SCID). This minimised the possibility of a link between *T. gondii* with impulsivity/aggression via the previously documented associations between *T. gondii* and psychopathology (described in more detail in Postolache et al. 2021), and between psychopathology and impulsivity / aggression.

## Aggression and impulsivity in healthy subjects in interactions with monoamine precursors

We further followed up with two “piggy-back” studies, one with measures of aggression and the other one with measures of impulsivity, measuring reciprocal moderations between *T. gondii* serointensity and seropositivity and KYN/TRP and phenylalanine/tyrosine ratios.

We reported a positive correlation between Phenylalanine/Tyrosine (Phe/Tyr) ratio and aggression, but only in *T. gondii* positive males (Mathai et al. 2016). In addition, in older *T. gondii* positive males with low (Phe/Tyr) ratio there was a decrease in aggression scores (Mathai et al. 2016). We also reported that in order that in order to score higher on impulsivity scores, a participant had to meet all three criteria: to be *T. gondii* positive, a younger man, and to be in the subgroup with high Phe/Tyr ratio (Peng et al. 2018).

Although these results may not be causal, or the consequence of “reverse causality”, the findings suggest a compelling hypothesis that elevated tyrosine levels may mitigate the effects of *T. gondii* seropositivity on trait impulsivity and aggressive behaviours. While a direct treatment of chronic toxoplasmosis presents challenges and potential adverse effects from long-term therapeutic intervention, tyrosine supplementation could offer an alternative approach. Specifically, tyrosine supplementation may effectively reduce the Phe/Tyr ratio, thus potentially neutralising the positive link between *T. gondii* positivity and elevated measures of impulsivity and aggression.

Given the favourable safety profile, widespread availability and cost-effectiveness of L-tyrosine supplementation, a randomised controlled trial investigating its efficacy in *T. gondii* positive individuals merits consideration. Such a study, focusing on subjects with elevated impulsivity, trait aggression and high phenylalanine/tyrosine ratios, represents an innovative research opportunity characterised by individual selection, minimal patient risk and moderate costs, while offering the potential for significant therapeutic benefits that *a priori* outweighs the substantial possibility of negative findings.

## Cats, pets, suicide risk and prevention

Although cats serve as definitive hosts in the transmission cycle of *T. gondii* to warm-blooded animals, and despite potential future evidence establishing a causal relationship between *T. gondii* serointensity and suicidal behaviour, in my clinical experience with individuals with higher potential for SSDV, I have observed that companion animals, particularly cats and dogs, play a substantial protective role against attempting suicide. The presence of dependents, most notably young children, represents one of the most robust protective factors against suicide. Similarly, pets fulfill multiple protective functions: they provide emotional comfort and companionship, mitigate loneliness – a well-established suicide risk factor – and, as a dependent, create a sense of responsibility and purpose that can serve as a powerful deterrent to suicidal behaviour.

Preventative measures such as limiting cats’ exposure to tissue cyst infected meat sources or contaminated food sources and promoting proper handling of cat litter remain important for infection control. Yet, the removal of a companion cat from an individual at elevated suicide risk, in particular if lonely and not having other dependents and positive reasons for living, could increase suicide risk. Concordantly, pet ownership may decrease suicide risk factors, of which most vital component is likely the deterrent of leaving behind a dependent who is unable to take care of himself/herself. There is a need for nuanced approaches in addressing both risks for *T. gondii* infection, as well as promoting protective factors in suicide risk management. For the multiple levels of complexity involving controlling *T. gondii* and its societal burden, broad transdisciplinary approaches and committed collaborations defined as a “One health” approach (Aguirre et al. 2019) are strongly needed.

In conclusion, our research team’s identification of the association between chronic “latent” *T. gondii* infection and suicidal behaviour emerged from analogical reasoning and several mistaken premises (such as assuming similarities of immune response to *T. gondii* as with many other parasites and allergens). While our initial premises were incorrect, they were “fertile errors” – they led to our productive investigations and discoveries. This phenomenon has been called “productive misconceptions” in the theory of science. Scientific progress often emerges not from perfect knowledge but from the creative tension between theory and observation, where discrepancies are becoming the seeds of new discovery.

Rigorous methodological development–facilitated by our collaborators’ expertise in animal studies, *post-mortem* analyses, *T. gondii* serology, and molecular neuroscience – helped us establish the foundation for our *T. gondii*/SSDV research program. This investigation has further evolved through multiple complementary layers of inquiry, encompassing macroepidemiology, microepidemiology, evolutionary theory, clinical observation, molecular mechanisms, cellular processes, *post-mortem* studies, and pharmacological investigations.

Three independent systematic reviews upheld our initial observation. However, compared to our other research endeavours, this work has presented unique challenges, particularly in two interrelated domains. The first concern was represented by our current inability to translate accumulated knowledge into improved health outcomes, enhanced longevity, better functioning and increased quality of life for seropositive patients, as therapeutic interventions for chronic toxoplasmosis have thus far yielded suboptimal results (Torrey 2024).

The second challenge involved the field’s ongoing struggle to definitively establish causality for the link between *T. gondii* infection and neuropsychiatric illness in human subjects. While the most direct approach would involve randomised trials of interventions that either cure chronic toxoplasmosis or prevent parasite reactivation, the absence of proven effective treatments has necessitated a focus on optimising existing psychotropic medications and psychotherapeutic approaches. Experimental infection in humans is a non-starter on ethical grounds, considering the practical impossibility to clear the infection, creating significant methodological constraints relative to self-limiting infections. We entertained the possibility to study effects of natural seroconversion in high-prevalence populations, such as the Old Order Amish, but realised that these studies will face currently unsustainable costs and limitations.

In my attempt not to shy away from the challenges, losses, redemptions and disappointments that have marked my *T. gondii*-SSDV journey, I hope our brief article resonates with its readers. Writing it induced a stirring nostalgia for the times when I innocently thought I firmly understood what I was doing and when clinical applications that would save lives seemed falsely within immediate reach. I retain the consolation that wrong premises, overgeneralised conclusions and inflated expectations, while initially serving as catalysts for discovery before becoming sources of embarrassment, end up as self-deprecating anecdotes for the ears of the few who will find them amusing and the minds of the very few who will find them inspiring.
